# Carboxylic Acid-Assisted Synthesis of Tin(II) Iodide: Key for Stable Large-Area Lead-Free Perovskite Solar Cells

**DOI:** 10.1021/acsenergylett.4c02027

**Published:** 2024-08-22

**Authors:** Wiktor Żuraw, Dominik Kubicki, Robert Kudrawiec, Łukasz Przypis

**Affiliations:** † Department of Semiconductor Materials Engineering, Wroclaw University of Science and Technology, Wybrzeze Wyspianskiego 27, 50-370 Wroclaw, Poland; ⊥ Saule Research Institute, Dunska 11, 54-427 Wroclaw, Poland; § Saule Technologies, Dunska 11, 54-427 Wroclaw, Poland; ∥ School of Chemistry, University of Birmingham, B15 2TT Birmingham, U.K.

## Abstract

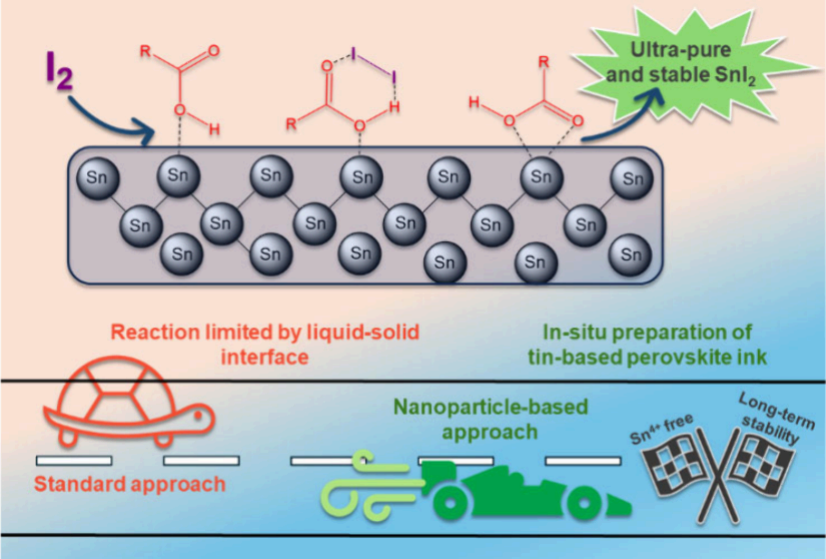

Despite significant progress in tin-based perovskites, the development of stable and high-performance tin-based perovskite solar cells (TPSCs) remains a challenge. In this pursuit, a multitude of strategies have been explored, encompassing the use of reducing agents, antioxidants, bulky cations, and customized solvent systems. We propose an improved approach for synthesizing SnI_2_ from elemental tin and iodine. Here, we generate tin nanoparticles grafted with a carboxylic acid *in situ* from tin powder–carboxylic acid-assisted synthesis (CAAS). This methodology not only improves the synthesis process of SnI_2_ but also enhances precursor stability against oxidation. We use ^119^Sn MAS NMR to study the atomic-level structure of the resulting FASnI_3_ thin films and find that the CAAS approach leads to highly pure and unoxidized material. We report remarkable reproducibility in fabricating large-area (1 cm^2^) flexible TPSCs with significant improvement in open-circuit voltage leading to the champion device showing a power conversion efficiency of 8.35%.

Metal-halide perovskites have emerged as game-changing materials for energy conversion. Their unique optoelectronic properties and straightforward fabrication processes hold great promise. These lightweight and cost-effective materials can be manufactured at high throughput using inexpensive raw materials and minimal energy inputs. Among solution-processable solar cells, lead-based perovskite solar cells are on the top with an impressive power conversion efficiency (PCE) of 26.1% for single-junction opaque solar cells.^1^ However, Pb toxicity poses a significant challenge for practical life applications. To address this problem, the most likely substitute is tin (Sn), which like Pb, is also a group 14 metal. In addition, Sn-based perovskites display similar or superior electronic and optical properties compared to Pb-based perovskites, such as higher charge carrier mobilities and long-lived hot carriers.^2^ The organic–inorganic tin-based perovskites show good semiconducting behavior with an optical bandgap in the range of 1.2–1.4 eV.^3−5^ The first investigation about their application in optoelectronic devices was reported in 2012 for CsSnI_3_.^6^ Since then, the development of tin perovskites has expanded to various optoelectronic fields, including photovoltaics, light-emitting devices, and photosensors.^7−10^ Despite these favorable optoelectronic properties, tin-based perovskite solar cells (TPSCs) still show PCEs that are much lower than those of their Pb counterparts. This is mainly attributed to the propensity of the metastable Sn^2+^ in the perovskite lattice to be oxidized to p-type Sn^4+^ defects in the presence of oxygen during the device fabrication (self-doping), or spontaneously through disproportionation in tin-poor environments.^11^ Therefore, stopping or controlling this oxidation pathway is one of the requirements to achieve efficient and stable TPSCs. For this reason, several strategies have been employed to tackle the oxidation of Sn^2+^. These include purifying or synthesizing high-purity SnI_2_ to minimize SnI_4_ content in the precursor,^12,13^ adding bulky A-site cations to stabilize the resulting films, or the use of new solvent systems to avoid oxidation by dimethyl sulfoxide (DMSO).^14−17^ To mitigate Sn^2+^ oxidation during fabrication processes, reducing agents or antioxidants are used. Several reducing agents have been implemented, including metallic Sn powder,^18^ hypophosphate,^19^ sodium borohydride,^20^ and various organic compounds.^21−24^ As for the antioxidants, the most extensively described is SnF_2_.^25^ Additionally, various sulfur organic derivatives have been used for this purpose.^26,27^ Most of these approaches are increasingly used simultaneously to fabricate high-performing TPSCs (Figure 1).

**Figure 1:**
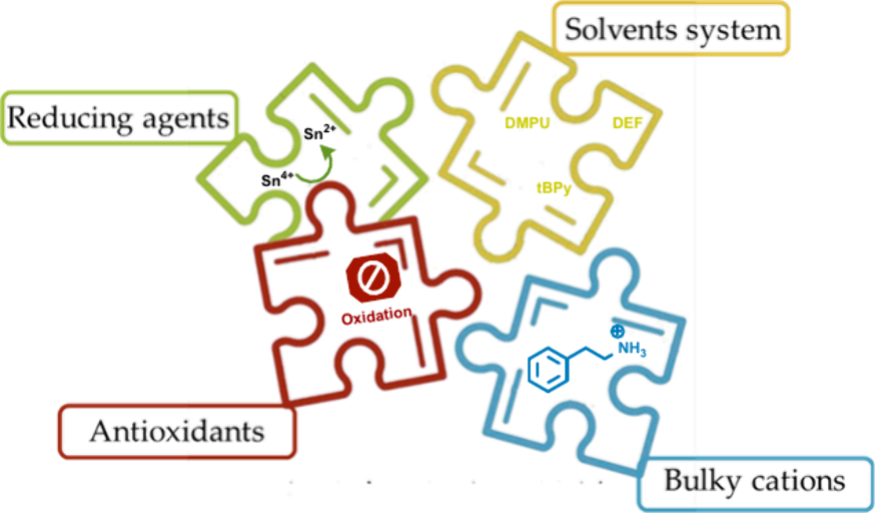
Advancing TPSCs: strategies for enhanced performance.

In this work, we present a novel approach for the synthesis of ultrapure tin(II) iodide, a critical component in the fabrication of TPSCs. Our methodology involves the surface functionalization of tin nanoparticles (NPs) with carboxylic acid ligands, dubbed carboxylic acid-assisted synthesis (CAAS). This method is not only aimed at synthesizing tin(II) iodide but also at hindering the oxidation process. The incorporation of carboxylic acid ligands serves a dual purpose. We expect a synergistic effect wherein these ligands positively interact with the tin-based perovskite compound during the crystallization stage. This interaction is pivotal for the formation of a stable perovskite structure. To study the influence of CAAS on the device performance, we fabricated flexible perovskite solar cells with an active area of >1 cm^2^. The champion device exhibits a PCE of 8.35%, with an open-circuit voltage (*V*_oc_) of 0.59 V, a short-circuit current density (*J*_sc_) of 21.60 mA/cm^2^, and a fill factor (FF) of 66.5%.

The synthesis of SnI_2_ from elements has been reported in the literature,^28^ and the use of tin NPs to improve tin-based perovskite ink has also been demonstrated.^24^ In our study, by combining these methodologies, we have proposed a novel approach to synthesize SnI_2_ in order to obtain a stable tin-based perovskite ink that is not only more resistant to oxidation but also exhibits high device efficiencies. We start by inspecting the key stage of the SnI_2_*in situ* synthesis, the solid–liquid interface interaction of metallic tin and the I_2_·DMSO complex. This interface is the limiting factor for the reaction. Therefore, to fully control this reaction, it is important to optimize this step. To achieve this, we focused on customizing that interface by increasing the surface area to volume ratio. This approach aimed to overcome limitations and facilitate faster and more efficient synthesis of pure and stable SnI_2_. Metal NPs exhibit highly reduced sizes, resulting in significantly enhanced reactivity, ideal for promoting the desired reaction pathway. To achieve this goal, we applied grafted tin NPs for SnI_2_ synthesis. By combining the advantages of SnI_2_ synthesis from elements and *in situ* Sn-nanoparticle generation, we anticipate significant enhancement in the performance of tin-based perovskite inks. Moreover, due to the boosted reaction rate, this method enables the production of SnI_2_ in a variety of solvent systems and provides the possibility to work in noninert atmospheres. This opens doors to improved lead-free perovskite solar cell technologies.

The most comprehensive methodology for the preparation of metal NPs involves treatment assisted with ligands.^29−31^ We chose to use carboxylic acids as ligands capable of modifying the tin surface. Moreover, carboxylic acids were reported in the literature as effective additives that aid in the formation and crystallization of perovskites.^32−36^ Therefore, in our concept, carboxylic acid serves not only as an agent for the formation of Sn-NPs for the synthesis of tin(II) iodide but also can positively affect perovskite formation.

We started the verification of the hypothesis about the key role of nanoparticles by looking for an approach that would involve the formation of Sn-NPs. In general, carboxylic acid can form stabilizing interactions with tin in three different ways.^37^ The first is dipole attraction (**I**), where the −OH group from a carboxylic acid interacts with metallic tin, which being a strong Lewis acid, has a strong affinity for groups containing oxygen. Another configuration is carboxylic acid acting as a pincer ligand (**II**), where the negative dipole moment is shared between two oxygen atoms. The last option combines the first two, forming a bridge-type interaction (**III**) where one carboxylic unit interacts with two tin centers (Figure 2a).

**Figure 2:**
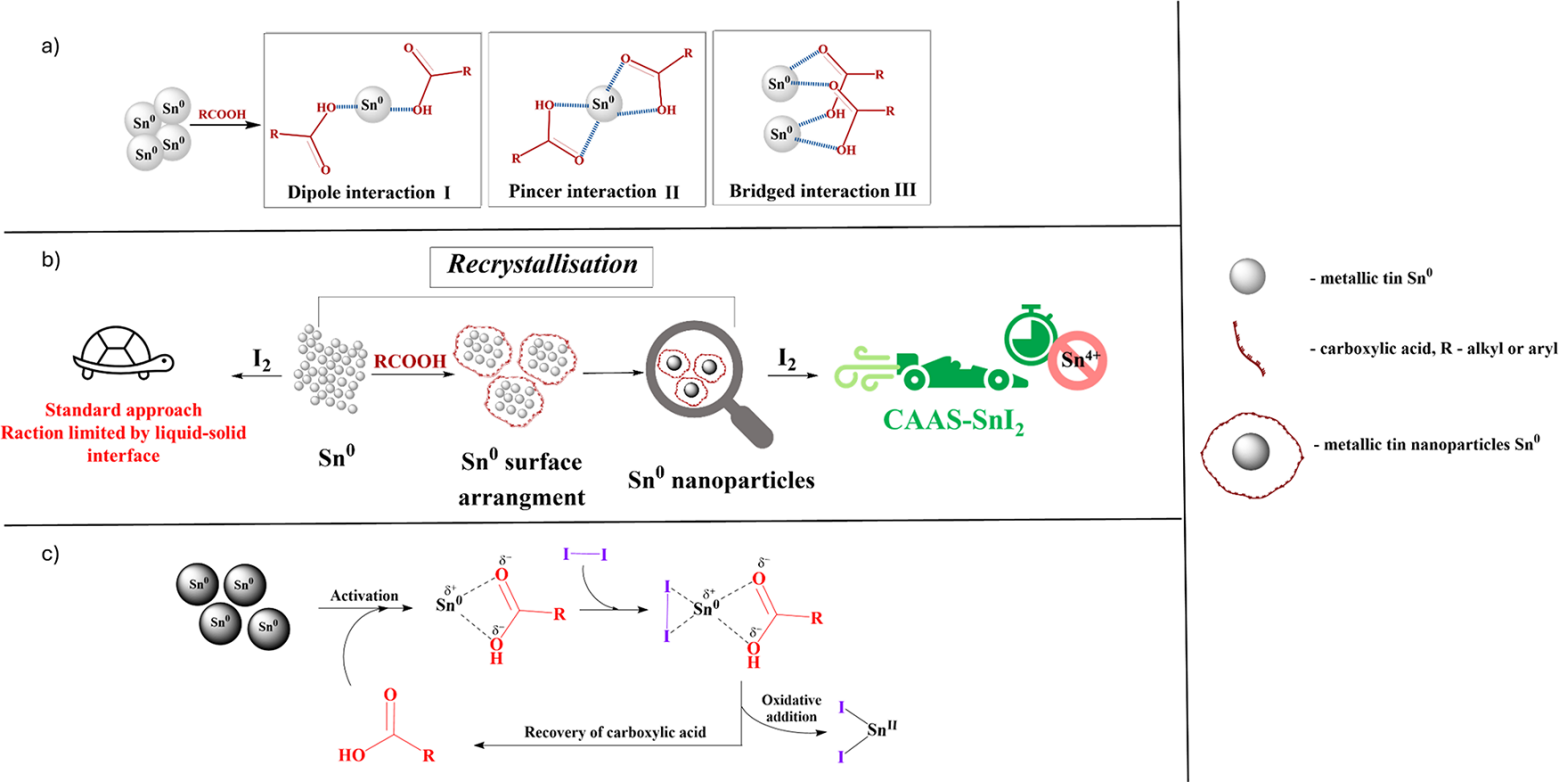
(a) Grafting of tin NPs with carboxylic acid—potential interactions. (b) Comparison of reactions with tin powder vs CAAS-SnI_2_. (c) Plausible mechanism of tin(II) iodide formation catalyzed by carboxylic acid.

In the CAAS approach, interactions of the carboxylic acid with tin are enough to form Sn-NPs and recrystallize the surface of tin powder (Figure 2b and Figure S2). Additionally, the anchored carboxylic acid units on the reaction surface can interact positively with iodine molecules, further enhancing the progress of the reaction. In light of the above facts, the natural choice was formic acid, the simplest carboxylic acid. Many preparation protocols for metallic nanoclusters using formic acid were reported in the literature.^38−40^ In further experiments, we focus on this acid to develop the perovskite ink preparation procedure. However, for a broader evaluation, we showed that the synthesis of SnI_2_ based on the CAAS approach is possible using different carboxylic acids (typical reducing agents or with additional functional groups), which opens the door to introducing additives tailored to the desired composition (Supplementary Discussion 1).

Based on these observations, we propose a plausible pathway for SnI_2_ formation in the CAAS process (Figure 2c). In the first step, carboxylic acid coordinates with metallic tin, forming a carboxylic acid-tin complex. In Figure 2a, we present three possible interactions for the carboxylic acid to the tin atom: dipole, pincer, and bridged interaction. However, it is more likely that the carbonyl oxygen of the carboxylic acid coordinates with the tin atom due to its higher nucleophilicity, making the pincer form of the complex the most probable. In the next step, an iodine molecule coordinates with the tin-carboxylic acid complex. The carboxylic acid facilitates the oxidative addition of iodine to the tin atom, forming SnI_2_ and regenerating the carboxylic acid for the next catalytic cycle. This proposed stabilization mechanism would explain the observed faster and more efficient reaction under carboxylic acid treatment. We expect that carboxylic acid will have a positive effect on perovskite crystallization. Moreover, the metallic tin NPs suppress the formation of Sn^4+^ ions through the reaction Sn^0^ + Sn^4+^ → 2Sn^2+^.

In our approach, we observed that the reaction proceeded at a faster rate compared to the protocol previously reported in the literature (standard synthesis)^28^ – in our method, the formation of SnI_2_ happened immediately. The comparison of the reaction rates between these methods is shown in Figure S3. The enhanced reaction facilitated by Sn-NPs offers numerous advantages for tin-based perovskite ink preparation, including easy scalability for large-scale production and the preparation of SnI_2_ in a variety of solvents (Figure S4). Moreover, our method does not require highly restrictive conditions (Figure S5), making it more practical for large-scale production. To evaluate the stability of our ink, we conducted an aging test under controlled conditions (Figure 3). After 2 h, the CAAS-SnI_2_ solution maintained its vibrant yellow color without any signs of aging, in contrast to the control (commercial SnI_2_) sample which promptly turned red. Based on these results, we conclude that, in line with our initial assumptions, the CAAS ink is more resistant to oxidation. Remarkably, these perovskite inks show no signs of aging after 2 years of storage in an N_2_-filled glovebox (Figure S6).

**Figure 3:**
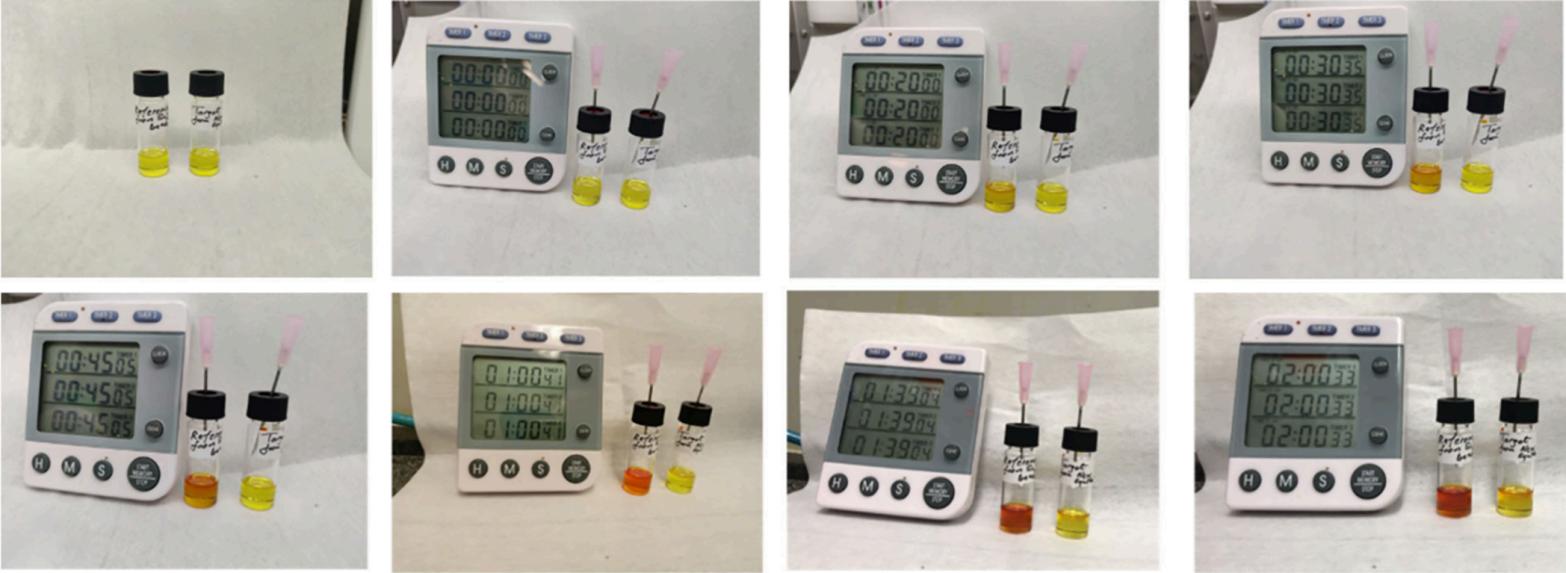
Images of SnI_2_ precursor solution in DMF:DMSO for different times of exposure to the air: control (on the left) and CAAS-SnI_2_ (on the right).

Next, we investigated how CAAS influences perovskite film formation. We prepared perovskite precursor solutions by mixing CAAS-SnI_2_ (target) and commercial SnI_2_ (control) in DMF:DMSO with FAI and SnF_2_ in a 1:1:0.1 molar ratio. Using the spin-coating technique with antisolvent approach, we fabricated highly reproducible uniform perovskite films. We analyzed the composition of perovskite layers using X-ray diffraction (XRD) and did not observe peaks corresponding to the 2D perovskite structure or additive (Figure S7). In the next step, we characterized the morphology of films using a scanning electron microscope (SEM). We confirmed a large grain size that was tightly packed in the film (Figure S8). The photoluminescence (PL) spectrum shown in Figure S9 displays higher emission for the CAAS-FASnI_3_ layer than for control FASnI_3_. These results indicate that the CAAS method enables the formation of high-quality perovskite films.

We next study the atomic-level structure of the material using solid-state NMR. ^119^Sn Magic Angle Spinning (MAS) NMR has been shown to be highly sensitive to the Sn oxidation state in halide perovskites in solution^41^ and the solid state.^42^ Notably, the solid-state spectrum of the Sn^2+^ perovskite species is sensitive to disproportionation (self-doping) with materials prepared under reducing conditions giving rise to narrow signals and the signal substantially broadening when the material is exposed to air (Figure 4a, middle spectra).^43^Figure 4b shows ^119^Sn MAS NMR spectra of a sample fabricated using the one-step antisolvent CAAS approach. The spectra show only the presence of FASnI_3_ whose signal is narrow (85.2 ± 0.8 ppm) and comparable to that previously reported for MASnI_3_ prepared in the presence of strongly reducing H_3_PO_2_. There are no detectable signals of metallic tin and FA_2_SnI_6_. These results indicate that the material is fully in its Sn^2+^, unoxidized form. ^13^C MAS NMR spectra of the material show the presence of the formate (C=O) and ethylenediammonium signals (Figure 4c), used as additives in the fabrication process, and residual DMSO, indicating that these species are preserved in the solid material after thin film fabrication. Cross-polarization (CP) and echo spectra are qualitatively similar to CP preferentially enhancing rigid local environments of the sample.

**Figure 4:**
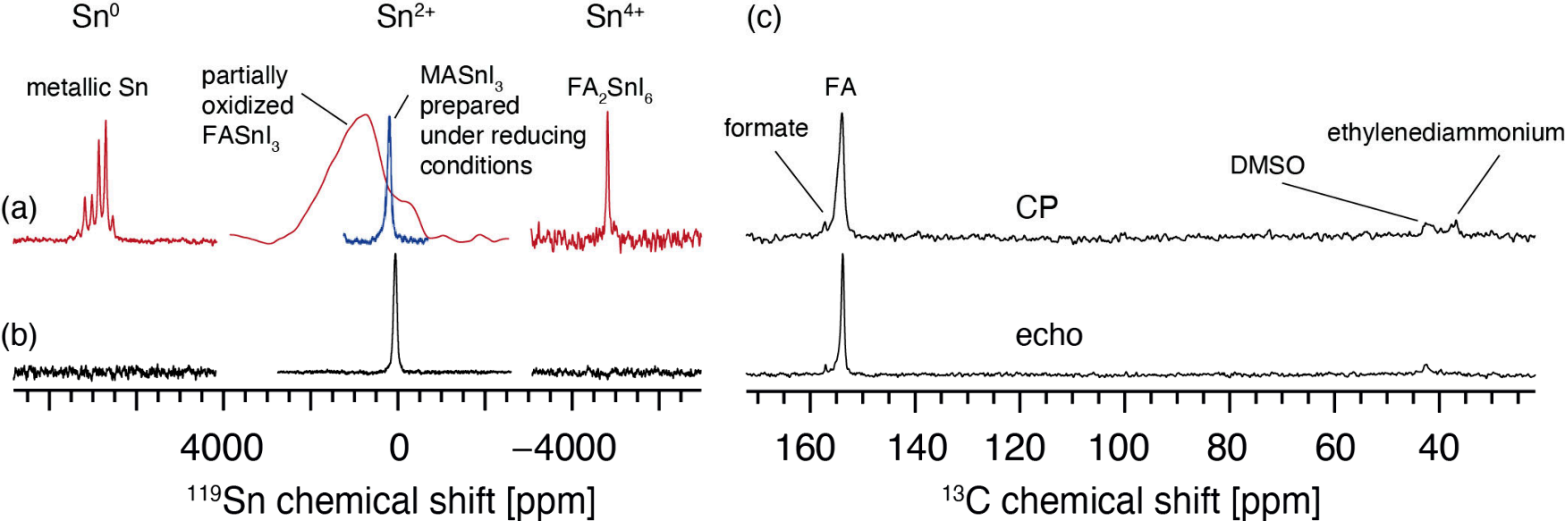
^119^Sn solid-state MAS NMR spectra of (a) reference materials, metallic tin, FASnI_3_ prepared without a reducing agent, MASnI_3_ prepared with a reducing agent (H_3_PO_2_), FA_2_SnI_6_ (data adapted from refs (42), red, and (43), blue), and (b) FASnI_3_ based on CAAS-SnI_2_. The T_1_ of this species is 0.45 s. (c) ^13^C solid-state MAS NMR spectra of FASnI_3_ based on CAAS-SnI_2_. (Data in panels b and c recorded at 11.7 T, 20 kHz MAS and 298 K.)

To study the influence of CAAS-SnI_2_ on device performance, we fabricated large-area (active area of 1 cm^2^) flexible perovskite solar cells with the simple perovskite composition and device structure: PET/IZO/PEDOT:PSS/FASnI_3_/C_60_/BCP/Ag. Ethylenediammonium diiodide (EDAI_2_) was used as an additive in perovskite precursor solution as a commonly known compound in TPSCs which improves reproducibility and device performance.^44^ We observed a significant increase in *V*_oc_ and thus PCE for devices made from CAAS-SnI_2_ compared to commercial SnI_2_ (Figure 5a). This is consistent with the ssNMR and PL results and is ascribed to a reduced defect density due to a decreased amount of Sn^4+^ impurities which play the role of nonradiative recombination centers.^45^ We note that many factors can influence *V*_oc_ and lower values compared to state-of-the-art can result from the large-area flexible substrate and simple 3D perovskite composition without any passivation layers.^46,47^ Short-circuit current was similar for both approaches and was mainly in the range of 18–20 mA/cm^2^ (Figure 5a). External quantum efficiency (EQE) spectra did not show considerable differences between both methods. Maximum EQE up to 77% was obtained for 510 nm and integrated *J*_sc_ matches with *J*_sc_ obtained from current density–voltage (J-V) scan (Figure 5b). The thickness of both perovskite layers was the same and reached 190 ± 10 nm (Figure S10). Additionally, CAAS showed better reproducibility of prepared PSCs with an average PCE of 7.17 ± 0.15%, compared to the average PCE of 6.07 ± 0.51% for PSCs made from commercial SnI_2_.

**Figure 5:**
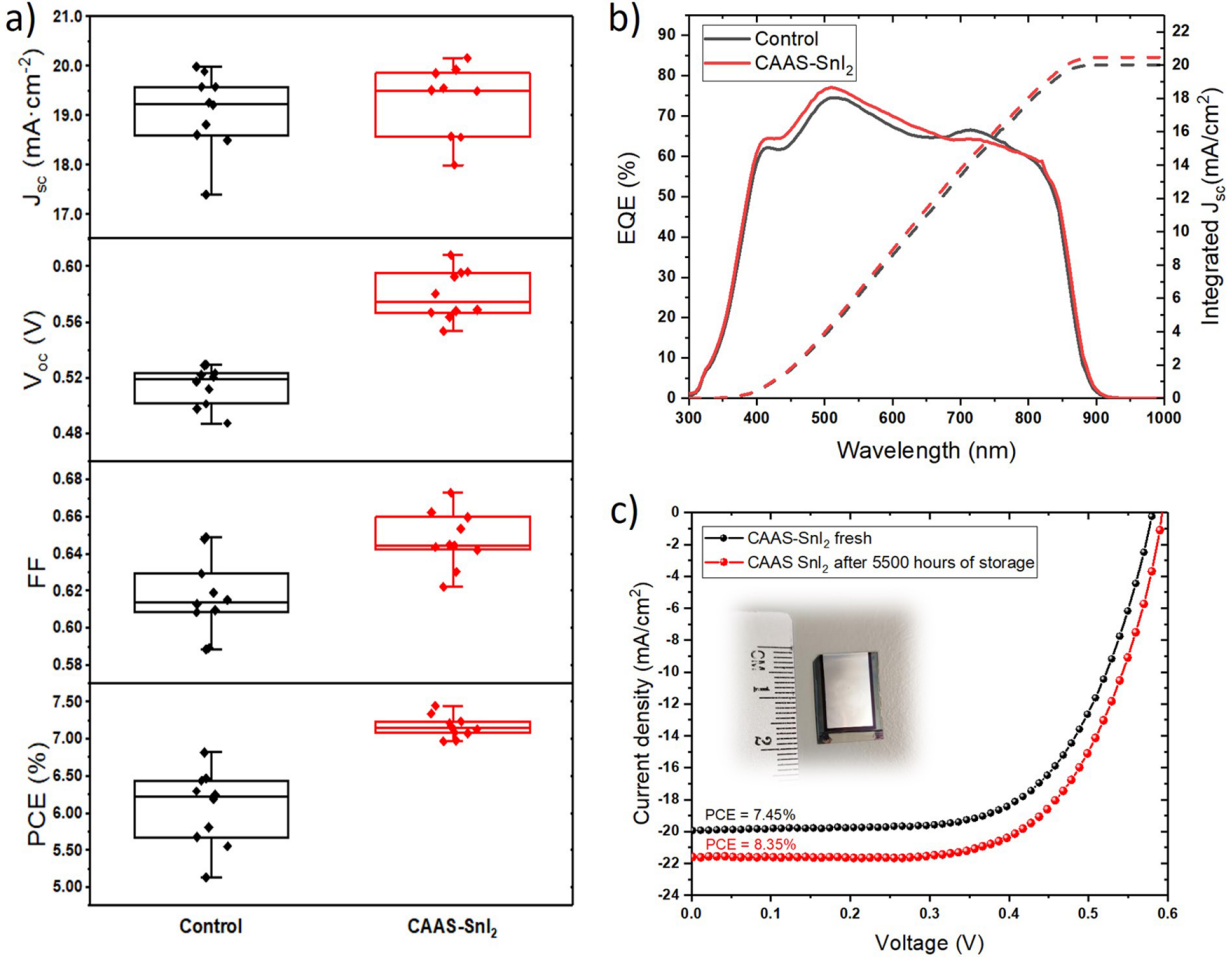
(a) J-V parameters and (b) EQE spectra of TPSCs for CAAS-SnI_2_ and commercial SnI_2_ (control). (c) J-V reverse scan for fresh and aged champion device (inset: picture of the flexible TPSC).

To study the charge carrier transport in prepared TPSCs, we measured dark J-V characteristics shown in Figure S11. The device with the CAAS layer exhibited a lower dark current density which can be attributed to the lower density of bulk or interface defect states. The relationship between reverse saturation dark current density (J_0_) and *V*_oc_ is given by *V*_oc_ = ,^48^ where *n* is an ideality factor, *k* is a Boltzmann’s constant, *T* is an absolute temperature and *q* is an elementary charge. The lower *J*_0_ obtained for CAAS leads to higher *V*_oc_ which agrees with the *V*_oc_ values obtained from J-V light measurements.

We also assessed the stability of unencapsulated PSCs inside an N_2_-filled glovebox. In the literature, it was reported that EDAI_2_ causes slow relaxation of the perovskite structure resulting in increasing performance in time with maximum PCE after 1–3 months of storage.^49^ We expected that effect in our PSCs but to avoid the influence of oxygen and water during J-V measurements in ambient conditions, we remeasured the champion device after 7 months of storage and we obtained PCE of 7.96%. Surprisingly, after 2 weeks PCE increased up to 8.35% which is the highest reported PCE for flexible lead-free PSC with a large active area (Figure 5c). That result also indicates that exposing devices to ambient conditions for a few minutes during J-V measurements can accelerate the passivation and crystal relaxation effect of EDAI_2_. PV parameters for the champion cell and record results from the literature are summarized in Table S1. The stability of the device under ambient conditions is presented in Figure S12. After 2000 h of storage on air (35–40% RH) the prepared device (without any encapsulation or passivation layer) retained 40% of the initial PCE.

In summary, we have introduced a novel method for the synthesis of ultrapure and stable SnI_2_, using a nanoparticle-based approach with carboxylic acid ligands (CAAS-SnI_2_). This innovative method involves nanoparticle surface functionalization, which we have demonstrated using various carboxylic acids, with formic acid showing the most promising results. The absence of Sn^4+^ species and the long-term stability of the SnI_2_ ink were confirmed through aging tests. ^119^Sn solid-state MAS NMR analysis revealed that this approach effectively eliminates self-doping, with the FASnI_3_ prepared in this way being free of Sn^4+^. This method serves as a versatile platform for the *in situ* preparation of tin-based perovskite ink. The fabricated large-area (1 cm^2^) flexible TPSCs achieved a remarkable PCE of 8.35%. These findings not only advance lead-free perovskite solar cell technology but also pave the way for scalable production of high-performance devices.

## Supporting Information Available

Experimental details including materials, synthesis, solar cells fabrication, characterization data (XRD patterns, SEM images, PL spectra), photographs of synthesized SnI_2_ and FASnI_3_ perovskite inks, additional discussion about interaction and reaction progress with different carboxylic acids, and JV parameters of state-of-the-art large-area spin-coated tin-based perovskite solar cells (PDF)
