# Supplementary material for: Carboxylic Acid-Assisted Synthesis of Tin(II) Iodide: Key for Stable Large-Area Lead-Free Perovskite Solar Cells
Source: ACS Energy Lett. 2024 Aug 22;9(9):4509–15. doi: 10.1021/acsenergylett.4c02027 (PMC11406576; doi:10.1021/acsenergylett.4c02027)
Supplement: Supplementary file 1 — nz4c02027_si_001.pdf [file nz4c02027_si_001.pdf]

Supporting information:

Carboxylic Acid-Assisted Synthesis of Tin(II) Iodide: Key for Stable Large-Area Lead-Free Perovskite Solar Cells

*Wiktor Żuraw[1,2,3]‡, Dominik Kubicki[4], Robert Kudrawiec[1] and Łukasz Przypis[1,2,3]‡**

[1]Department of Semiconductor Materials Engineering, Wroclaw University of Science and Technology, Wybrzeze Wyspianskiego 27, 50-370 Wroclaw, Poland

[2]Saule Research Institute, Dunska 11, 54-427 Wroclaw, Poland

[3]Saule Technologies, Dunska 11, 54-427 Wroclaw, Poland

[4]School of Chemistry, University of Birmingham, B15 2TT Birmingham, U.K.

‡These authors made equal contributions to the work

*Corresponding author: Lukasz.Przypis@pwr.edu.pl

Experimental Section

Materials:

Tin powder (99.8%, Thermo Scientific, 325 mesh), iodine 99.5% (Sigma-Aldrich), formic acid 99% (Thermo Scientific), tin(II) iodide (anhydroBeads™ 10 mesh, 99.99% trace metals basis, Sigma-Aldrich), formamidinium iodide (FAI, 99.9%, Ajay North America), tin(II) fluoride (SnF_2, 99%, Sigma-Aldrich), ethylenediammonium diiodide ($EDAI_2$, >98.0%, Sigma-Aldrich), poly(3,4-ethylenedioxythiophene) polystyrenesulfonate (PEDOT:PSS, Clevios Al 4083 dispersion), fullerene-C_{60} (99.9%, Sigma-Aldrich), bathocuproine (BCP, 99.99%, Sigma-Aldrich), silver (99.99%, Kurt. J. Lesker), N,N-dimethylformamide (DMF, 99.8%, Sigma-Aldrich), dimethyl sulfoxide (DMSO, 99.9%, Sigma-Aldrich), toluene (99.8%, Sigma-Aldrich) were used without further purification. Indium zinc oxide (IZO)-coated polyethylene

terephthalate (PET) substrates (sheet resistance of 15 $\Omega\cdot\square^{-1}$) were bought from Eastman Chemical Company.

Perovskite film fabrication:

Under a nitrogen atmosphere, the tin powder was suspended in 1 mL of DMF:DMSO 4:1 (v/v). Subsequently, 15 μl of formic acid was added, resulting in a greyish-dense dispersion. The dispersion was stirred for 4 hours. The 0.8 mmol of iodine was added gradually to the dispersion under vigorous stirring. The mixture was further agitated for 15 minutes and SnI_2 solution was obtained. The precursor solution for the $FASnI_3$ perovskite film was prepared by mixing SnI_2 ink with 0.8 mmol of FAI and 0.08 mmol of SnF_2. For solar cells fabrication, additionally, 2 mol% of $EDAI_2$ was added to the perovskite precursor solution. For comparison with commercial SnI_2, a „Control" solution was made by mixing SnI_2, FAI, SnF_2, formic acid, and tin powder (additionally $EDAI_2$ for solar cells) with the same molar ratio to form 0.8 M perovskite solution. Before deposition, the solution was filtered by a 0.22 μm PTFE filter. The perovskite precursor solution was spin-coated at 6000 rpm for 30 s with a ramping rate of 3000 $rpm\cdot s^{-1}$. Toluene was dropped onto the surface at the 10th second. The resulting film was annealed at 50 °C for 1 minute and at 100 °C for 10 minutes.

Solar cell fabrication:

Perovskite solar cells were fabricated on flexible polyethylene terephthalate (PET) foil. IZO-coated PET substrates were etched by dipping one side in the HCl solution (15 wt% in deionized water) and then cleaned by ultrasonication in deionized water and isopropanol for 10 minutes in each solvent and dried by nitrogen flow. After that, substrates were treated with oxygen plasma for 2 minutes. The PEDOT:PSS dispersion was spin-coated at 5000 rpm for 45 seconds, followed by annealing at 120 ℃ for 30 minutes. The perovskite films were deposited as mentioned above. Then 30 nm of fullerene C_{60} and 5 nm of BCP were sequentially evaporated on the perovskite layer. Finally, 100 nm of silver electrode was deposited on top of devices by thermal evaporation through a shadow mask resulting in an active area of >1 cm^2.

Characterization:

Solid-state NMR

Solid-state MAS NMR spectra of ^{119}Sn (186.5 MHz) were recorded on a Bruker Avance Neo 11.7 T spectrometer equipped with a 3.2 mm MAS probe using 154 kHz (^{119}Sn) and 62.5 kHz (^{13}C) RF strength. The recycle delays were as follows: for ^{119}Sn, 5 ms for the metallic tin and FA_2SnI_6 regions, and 1 s for the $FASnI_3$ region, in accordance with reference[1]; for ^{13}C echo, 10 s, for ^{1}H-^{13}C CP, 15 s. Number of scans: for ^{119}Sn, 30000 (metallic tin region), 25600 (FA_2SnI_6 region), 1664 for the $FASnI_3$ region; for ^{13}C, 4096 (echo), 2712 (CP). ^{119}Sn chemical shifts were referenced to neat SnO_2 at -604 ppm. ^{13}C chemical shifts were referenced to the CH carbon (38.48 ppm) of neat adamantane.

Current-voltage measurements

Current density-voltage measurements were carried out by a Keithley 2461 source measure unit. The solar cells were illuminated under ambient conditions with a simulated AM1.5G irradiation of 100 $mW \cdot cm^{-2}$ using an AAA-rated solar simulator obtained from Abet Technologies, sun 2000 which was calibrated against an RR-208-KG5 silicon reference cell also procured from Abet Technologies.

X-ray diffraction measurements

X-ray diffraction (XRD) patterns were collected with Rigaku MiniFlex600 (Cu Kα radiation, λ=1.5406 Å) diffractometer. The samples were measured using θ-2θ scans. The setup was equipped with a copper anode and a graphite monochromator to select Cu Kα radiation (2θ 5–50 deg; the diffractograms were scanned using 0.1 deg steps and a counting time of 1 s per step).

Scanning electron microscopy

Top-view morphology images of samples were obtained with a field emission SEM (Phenom ProX), which had an accelerating voltage of 10 kV with a working distance of 8.0 mm. Cross-section images were developed by deploying a focus ion beam scanning electron microscope, FIB-SEM (FEI Helios 600), which had an accelerating voltage of 2 kV.

Photoluminescence measurements

Photoluminescence measurements were performed with the Photon Etc IMA-VIS setup. A green laser (wavelength: 532 nm) was used as the excitation source.

External quantum efficiency

External quantum efficiency measurements were obtained with the Bentham PVE300 photovoltaic QE system.

Supplementary Discussion 1:

In addition to the simple formic acid (ForA), we also tested oxalic acid (OxA, closely resembling ForA), citric acid (featuring additional oxygen atoms, CitA), 2,3-pyrazinedicarboxylic acid (with nitrogen atoms, PDCA), and 2,5-thiophenedicarboxylic acid (incorporating an extra sulfur atom, TDCA). Heteroatoms in those acids (such as O, N, S) act as Lewis bases, conceivably leading to additional interactions with the tin metal surface. These interactions have the potential to influence the formation of SnI_2 and the properties of the final perovskite ink. Furthermore, our selected additives can be classified into two categories: reducing agents and compounds with strong coordination abilities to metals (**Figure S1a**). This distinction was crucial as we aimed to discern which factor plays a more significant role in the formation of SnI_2. Firstly, we suspended the tin powder in DMF:DMSO 4:1 (v/v) solution and then we added carboxylic acid (**Figure S1b**). Except PDCA, for other dispersions we got grey-milky color. The tin powder appeared to be uniformly distributed within the solvent mixture. For the PDCA, we obtained a yellow dispersion which we attribute to the oxidation of Sn^0 to Sn^{2+} ions by this acid. PDCA can also strongly coordinate with metal ions resulting in a polymeric structure.[2] In the next step, tin powder activated in this way was reacted with iodine beads (**Figure S1c**). The reaction was fastest for ForA, and also immediate effects were observed for TDCA and CitA – we obtained yellow solutions indicating that all of the iodine has reacted. The reaction was slower with OxA and PDCA. However, with OxA, tin was in the end converted to SnI_2. The observation of the orange-red color in the PDCA system indicated potential further evolution to Sn^{4+}, which can strongly interact with PDCA.[2] To confirm the identity of the compound formed in this case, a simple test was conducted by adding formamidinium iodide (FAI) to the ink. If SnI_2 was indeed formed, perovskite-complex should

have occurred in the presence of the organic component. However, no change in color to yellow was observed even after adding an equal volume of the organic component. The ink retained its intense red color for at least 24 hours. We concluded that the production of perovskite ink was not achievable in this system. In summary, the synthesis of SnI_2 based on CAAS is possible using the versatile nature of carboxylic acids. However, competitive complexation may occur if the carboxylic acid interacts too strongly with the tin ions, as observed with PDCA.

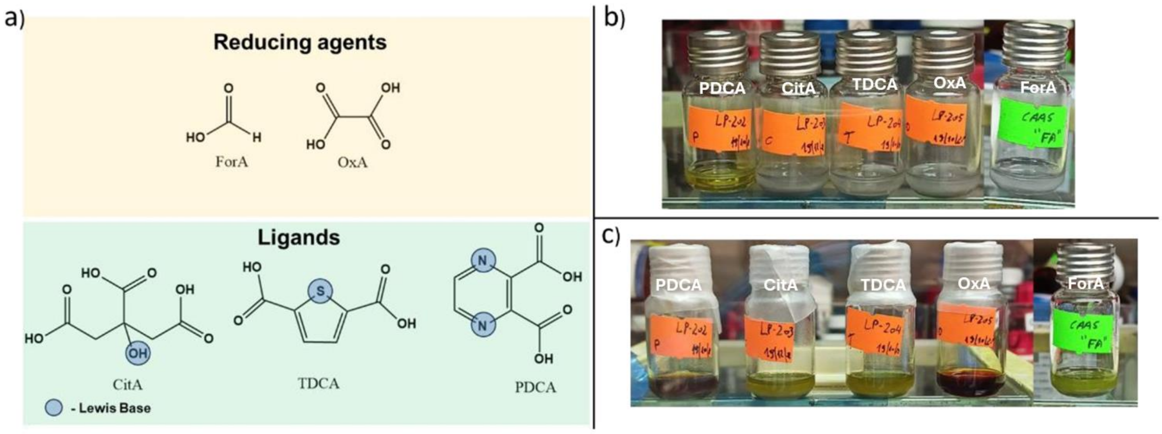

Figure S1. (a) Structures of tested carboxylic acids with highlighted additional functionalities; (b) Interaction of different carboxylic acids with tin powder; c) Reaction progress after adding iodine (after 5 minutes).

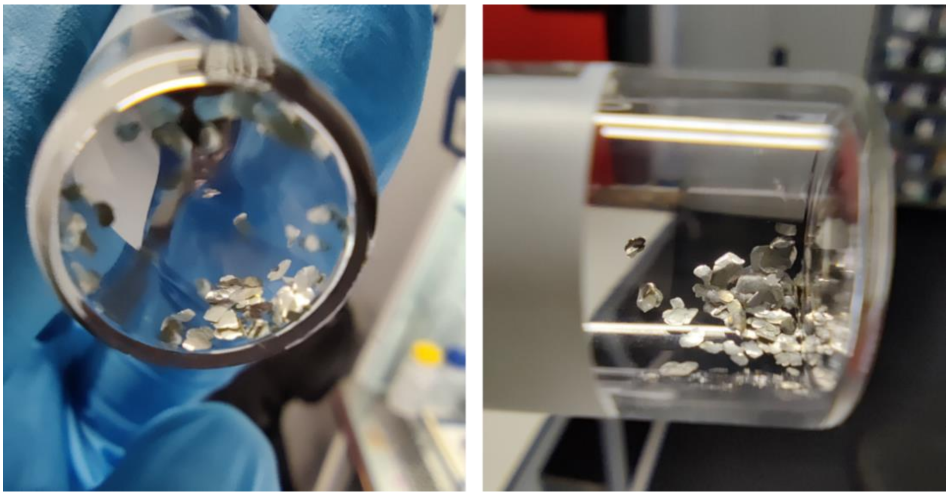

Figure S2. Tin metallic flakes collected after SnI_2 ink preparation.

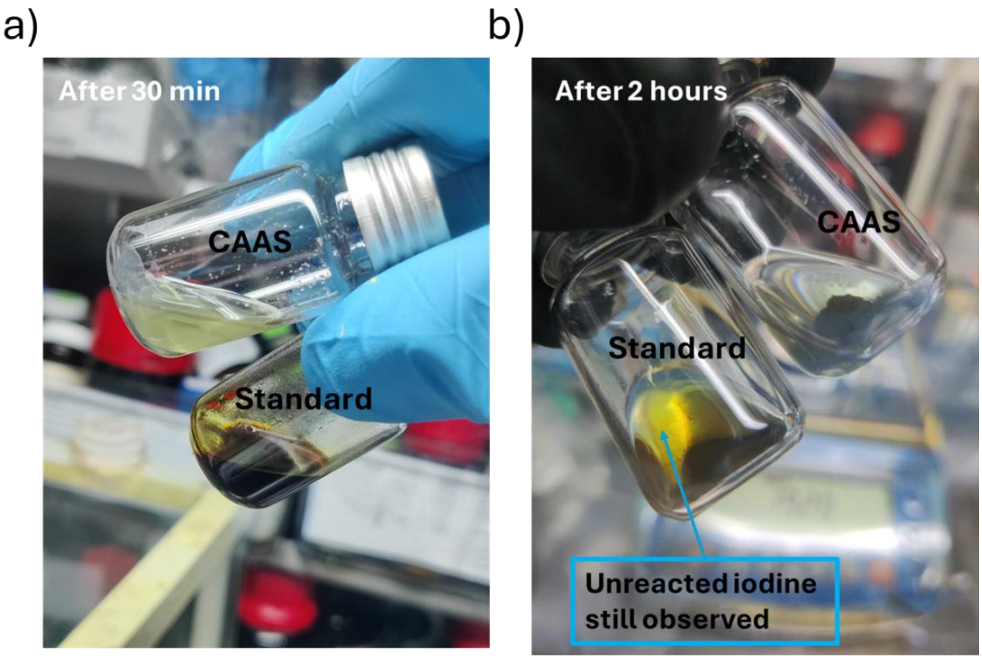

Figure S3. Comparison of SnI_2 synthesis progress over time a) 30 minutes; b) 2 hours - standard synthesis vs. CAAS method in DMSO.

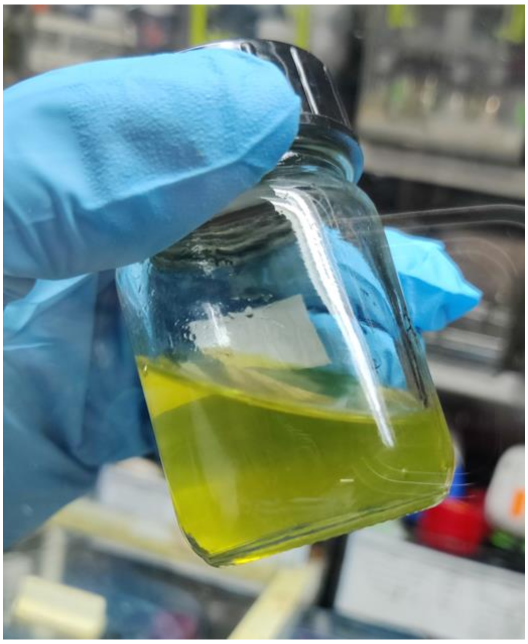

Figure S4. 20 ml of $FASnI_3$ ink prepared using the CAAS method.

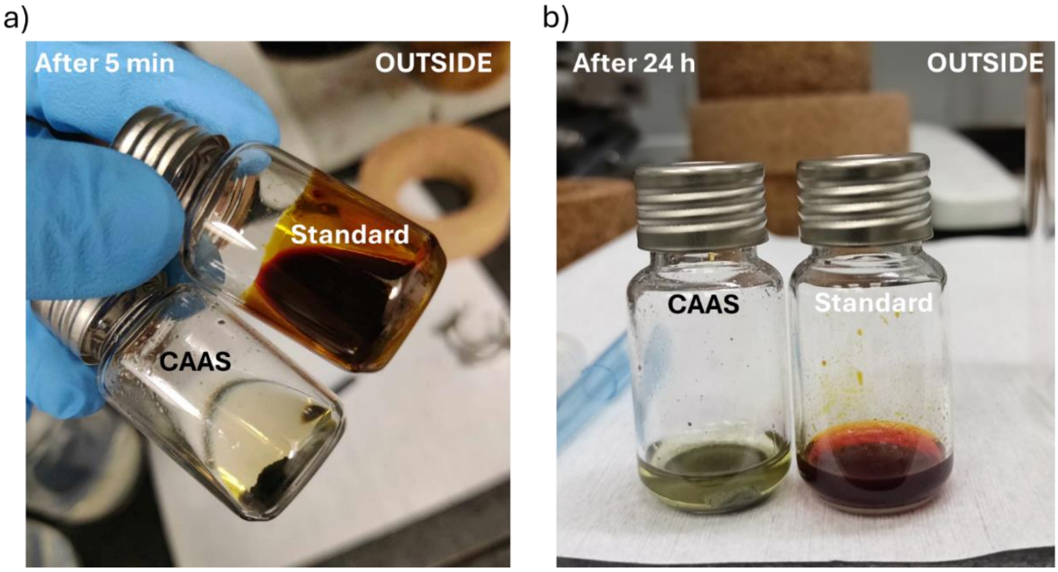

Figure S5. Comparison of SnI_2 synthesis progress over time under ambient conditions a) 5 minutes; b) 24 hours - standard synthesis vs. CAAS method in DMSO.

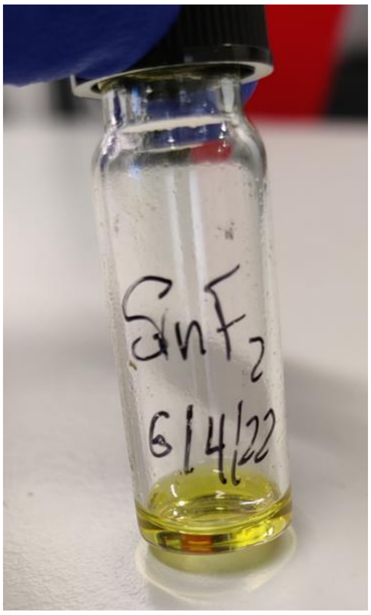

Figure S6. Two years old $FASnI_3$ ink stored in the glove-box.

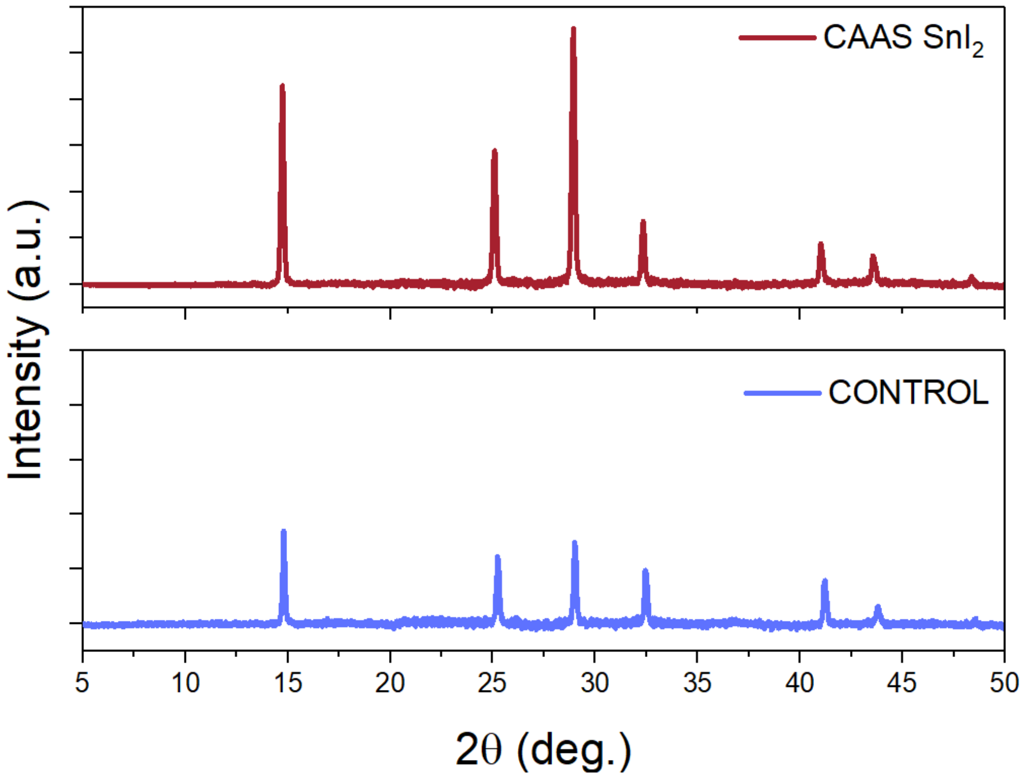

Figure S7. XRD of $FASnI_3$ films based on commercial SnI_2 (Control) and CAAS method.

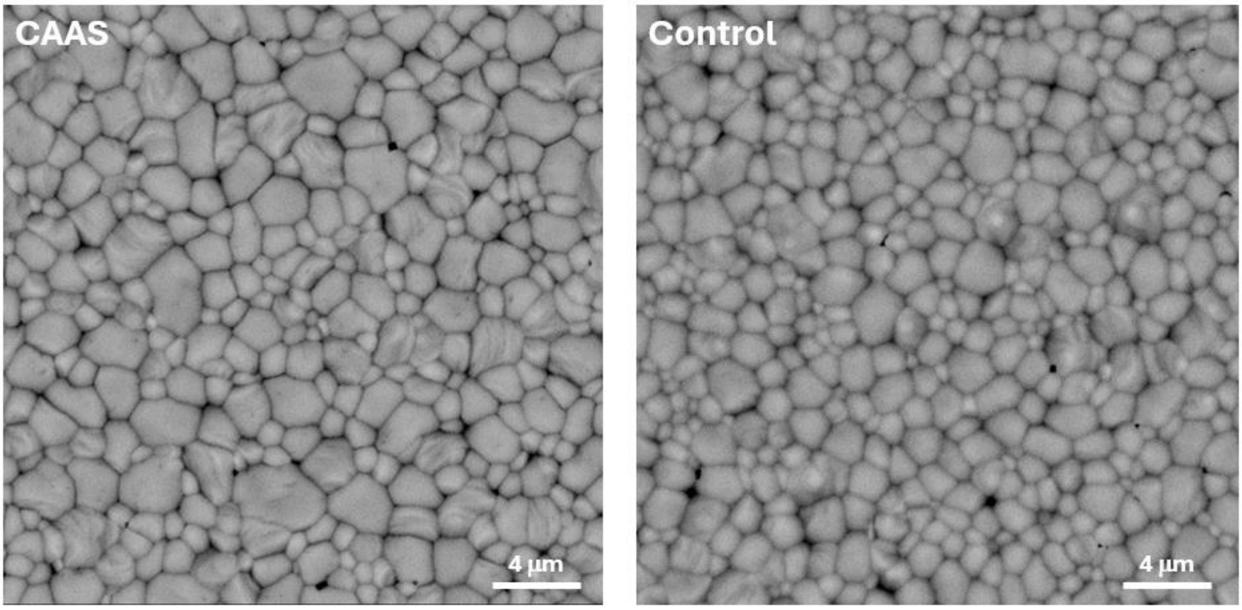

Figure S8. SEM images of $FASnI_3$ films based on commercial SnI_2 (Control) and CAAS method.

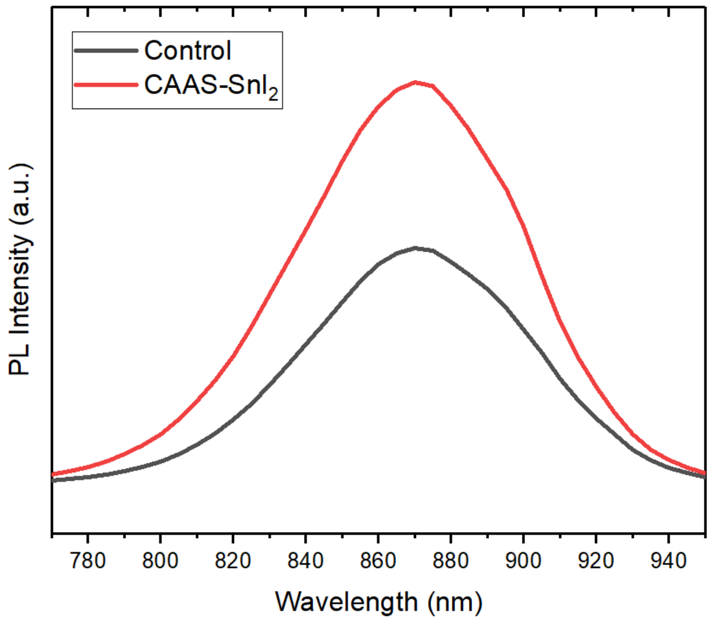

Figure S9. Photoluminescence spectra for control and CAAS-SnI_2 $FASnI_3$ layers.

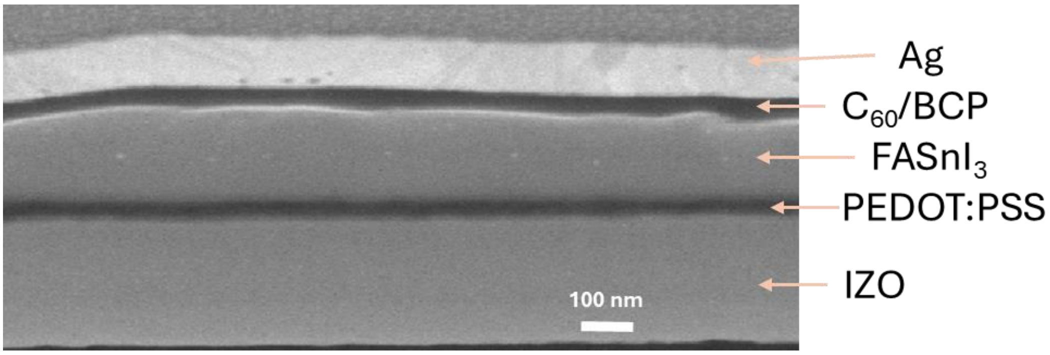

Figure S10. FIB-SEM image of the CAAS-SnI_2 TPSC.

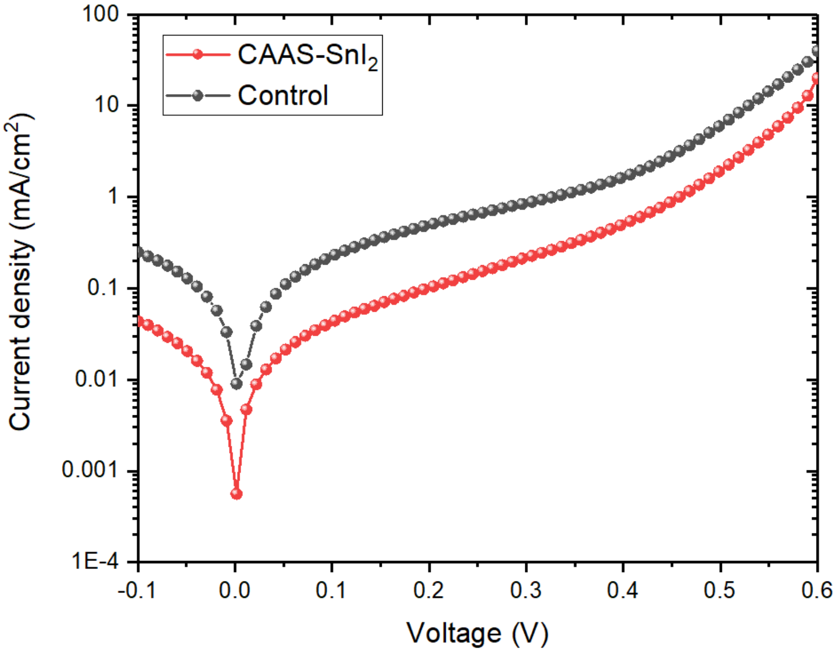

Figure S11. Dark J-V scans for control and CAAS-SnI2 TPSCs.

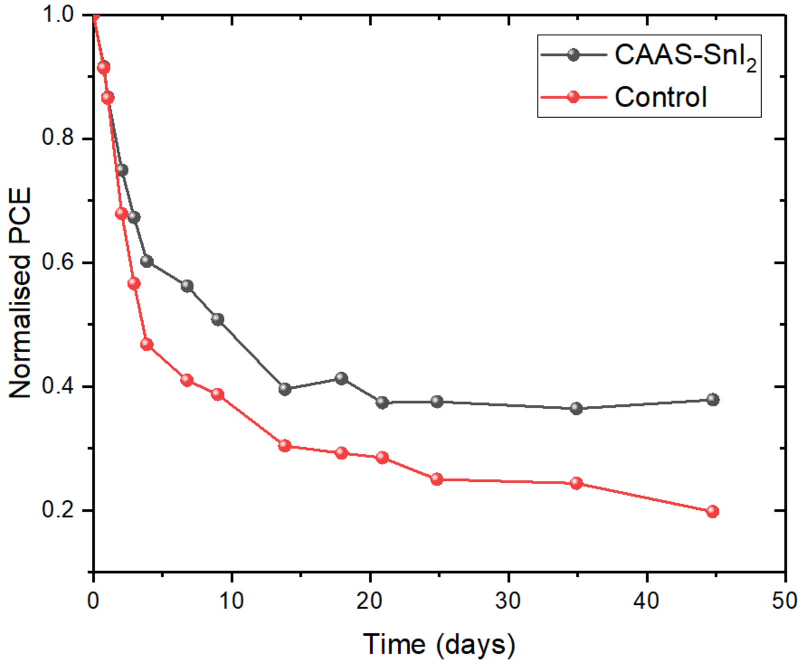

Figure S12. Stability of prepared TPSCs exposed to an ambient atmosphere (35-40% RH).

Table S1. State-of-the-art large-area spin-coated tin-based perovskite solar cells.

Substrate	Active area (cm^2)	J_{sc} (mA/cm^2)	V_{oc} (V)	FF (%)	PCE (%)	Strategy
Rigid	1.02	19.59	0.53	61	6.33	MACl vapor annealing[3]
Rigid	1	17.57	0.56	72.0	7.08	Conjugated organic cation additive[4]
Rigid	1	20.25	0.63	61.2	7.8	$CsSnI_3$ stabilizer[5]
Rigid	1	19.80	0.68	66.2	8.9	N-type additive[6]
Rigid	1.02	19.96	0.77	65.7	10.09	2-step deposition method[7]
Rigid	1.02	22.69	0.775	68.6	12.05	Modification of NiO_x/perovskite interface[8]
Flexible	1	19.98	0.56	58.8	6.6	$CsSnI_3$ stabilizer[5]
**Flexible**	**1**	**21.60**	**0.59**	**65.5**	**8.35**	**This work**

References:

(1) Kubicki, D. J.; Prochowicz, D.; Salager, E.; Rakhmatullin, A.; Grey, C. P.; Emsley, L.; Stranks, S. D. Local Structure and Dynamics in Methylammonium, Formamidinium, and Cesium Tin(II) Mixed-Halide Perovskites from 119Sn Solid-State NMR. *J Am Chem Soc* **2020**, *142* (17), 7813–7826. https://doi.org/10.1021/jacs.0c00647.

(2) Yin, H. D.; Li, F. H.; Wang, C. H. Syntheses, Characterization and Crystal Structure of Diorganotin and Triorganotin Heterocyclicdicarboxylates with Monomeric, 2D Network and 3D Framework Structures. *Inorganica Chim Acta* **2007**, *360* (8), 2797–2808. https://doi.org/10.1016/j.ica.2006.12.012.

(3) Chowdhury, T. H.; Kayesh, M. E.; Lee, J. J.; Matsushita, Y.; Kazaoui, S.; Islam, A. Post-Deposition Vapor Annealing Enables Fabrication of 1 Cm2 Lead-Free Perovskite Solar Cells. *Solar RRL* **2019**, *3* (12). https://doi.org/10.1002/solr.201900245.

(4) Ran, C.; Gao, W.; Li, J.; Xi, J.; Li, L.; Dai, J.; Yang, Y.; Gao, X.; Dong, H.; Jiao, B.; Spanopoulos, I.; Malliakas, C. D.; Hou, X.; Kanatzidis, M. G.; Wu, Z. Conjugated Organic Cations Enable Efficient Self-Healing FASnI3 Solar Cells. *Joule* **2019**, *3* (12), 3072–3087. https://doi.org/10.1016/j.joule.2019.08.023.

(5) Ye, T.; Wang, X.; Wang, K.; Ma, S.; Yang, D.; Hou, Y.; Yoon, J.; Wang, K.; Priya, S. Localized Electron Density Engineering for Stabilized Β-ΓCsSnI3-Based Perovskite Solar Cells with Efficiencies >10%. *ACS Energy Lett* **2021**, *6* (4), 1480–1489. https://doi.org/10.1021/acsenergylett.1c00342.

(6) Zhang, Z.; Su, Z.; Li, G.; Li, J.; Aldamasy, M. H.; Wu, J.; Wang, C.; Li, Z.; Gao, X.; Li, M.; Abate, A. Improved Air Stability of Tin Halide Perovskite Solar Cells by an N-Type Active Moisture Barrier. *Adv Funct Mater* **2024**, *34* (2). https://doi.org/10.1002/adfm.202306458.

(7) Liu, X.; Wu, T.; Luo, X.; Wang, H.; Furue, M.; Bessho, T.; Zhang, Y.; Nakazaki, J.; Segawa, H.; Han, L. Lead-Free Perovskite Solar Cells with Over 10% Efficiency and Size 1 Cm2 Enabled by Solvent–Crystallization Regulation in a Two-Step Deposition Method. *ACS Energy Lett* **2022**, *7* (1), 425–431. https://doi.org/10.1021/acsenergylett.1c02651.

(8) Li, B.; Zhang, C.; Gao, D.; Sun, X.; Zhang, S.; Li, Z.; Gong, J.; Li, S.; Zhu, Z. Suppressing Oxidation at Perovskite–NiOx Interface for Efficient and Stable Tin Perovskite Solar Cells. *Advanced Materials* **2023**. https://doi.org/10.1002/adma.202309768.
